# Aftersensations and Lingering Pain After Examination in Patients with Fibromyalgia Syndrome

**DOI:** 10.1093/pm/pnaf080

**Published:** 2025-08-06

**Authors:** Richard J Berwick, David A Andersson, Andreas Goebel, Andrew Marshall

**Affiliations:** Department of Pain Medicine, Pain Research Institute, University of Liverpool, Liverpool, UK; Department of Pain Medicine, Walton Centre, Longmore Lane, Liverpool, UK; Department of Pain Medicine, Wolfson Centre for Age-Related Disorders, King’s College London, Liverpool, UK; Department of Pain Medicine, Pain Research Institute, University of Liverpool, Liverpool, UK; Department of Pain Medicine, Walton Centre, Longmore Lane, Liverpool, UK; Department of Pain Medicine, Pain Research Institute, University of Liverpool, Liverpool, UK; Department of Pain Medicine, Walton Centre, Longmore Lane, Liverpool, UK

**Keywords:** Fibromyalgia, Chronic Pain, Painful Aftersensations, PAS, Quantitative Sensory Testing, QST, Lingering Pain

## Abstract

**Background:**

Fibromyalgia syndrome (FMS) is a chronic widespread pain condition with mixed peripheral and central contributions. Patients display hypersensitivities to a spectrum of stimuli. Patients’ blunt pressure pain thresholds are typically reduced, and sometimes (∼15%) gentle brushstroke induces allodynia. However, aftersensations after these stimuli have not, to our knowledge, been reported.

**Methods:**

We examined the perception of blunt pressure and “pleasant touch” in FMS. Patients were first interviewed and completed standard psychometric questionnaires. We then measured their sensitivity to blunt pressure and perception of pleasant touch, including aftersensations; patients were followed up for 5 days to evaluate lingering pain from blunt pressure.

**Results:**

We recruited 51 patients with FMS and 16 pain-free healthy controls (HCs) at a UK Pain Management Centre. Forty-four patients completed the aftersensation protocol. Most patients reported pain after the application of less mechanical pressure than the level of pressure at which HCs reported pain; median arm and leg thresholds for the patients with FMS were 167 kPa and 233 kPa, respectively. Eighty-four percent (31/37) of patients reported ongoing pain at the site of pressure application 1 day after testing, and 49% (18/37) still perceived pain at 5 days. Aftersensations after brushstroke were common in the FMS group, reported by 77% (34/44) of patients with FMS vs 25% (4/16) of HCs; 34% (15/44) of patients, but no HCs, perceived these aftersensations as uncomfortable. For patients with FMS who experienced aftersensations, brushstroke pleasantness ratings were reduced, and the skin was often an important site of pain.

**Conclusion:**

Pain after blunt pressure assessment typically lingers for several days. Aftersensations after brushstroke stimulation are a previously unreported FMS phenomenon. They are associated with tactile anhedonia and might identify a clinically distinct subgroup.

## Introduction

Fibromyalgia syndrome (FMS) is a chronic widespread pain condition of uncertain etiology.^1^ FMS pain, associated tenderness^1^, and fatigue often have a devastating impact on patients’ function, activity, and quality of life.^2^^,^^3^ FMS has been classically viewed as a pain state with central amplification.^1^^,^^2^^,^^4^ However, there is also mounting evidence of peripheral abnormalities, including small-fiber pathology with abnormal nociceptor function^5–9^ and abnormal thermoregulatory peripheral innervations.^10^

In patients with FMS, quantitative sensory testing (QST)^11^^,^^12^ demonstrates marked hypersensitivity to a broad spectrum of standardized stimuli; such hypersensitivity is established by registering the patient’s evaluation of the stimulus while it is applied.^13^^,^^14^ Patients’ blunt pressure pain thresholds are typically reduced, which is termed *static mechanical allodynia*^15^, but gentle brushstroke alone can induce pain*,* or *dynamic mechanical allodynia*, in 10–20%.^16^ In relation to noxious thermal stimuli (i.e.*,* stimuli that would be painful in normal skin), patients with FMS, similar to patients with other chronic pain conditions, can report painful aftersensations (PAS), i.e., sensations that persist even though application of the stimulus has ceased.^17–19^. In FMS, such PAS occur in up to 83% of cases after noxious thermal stimuli (e.g., 49.5°C to 51.5°C), with the remainder of patients experiencing nonpainful aftersensations.^20^ Thermal PAS also occur in healthy individuals at a frequency of 20% to 37%, but aside from being more common in FMS, they are more painful and longer-lasting in FMS.^20–24^ After noxious mechanical pressure cuff stimuli, PAS also occur at an increased frequency in FMS, at 50% in patients with FMS vs 12% in controls, and are correlated with clinical pain intensity.^25^

Anecdotally, other types of skin stimuli can also elicit painful aftersensations in patients with FMS; for example, in our practice, tender point testing required for the 1990 American College of Rheumatology^26^ (ACR) diagnostic protocol seems to cause long-lasting pain increases at the testing sites. To our knowledge, no quantitative or qualitative data on aftersensations after brushstroke stimuli have been published. Our goals in the present study were to describe, in patients with persistent FMS, aftersensations arising from aspects of mechanical QST assessment and from clinical examination as per ACR 1990, as well as to characterize subgroups formed on the basis of these phenomena. Here, we report on aftersensations after application of both blunt mechanical pressure and brushstroke in a cohort of patients with FMS.

## Methods

### Study Design and Study Subjects

Patients participated in an ongoing phenotyping study aiming to correlate clinical with immunological phenotypes (ISRCTN: 18414398). They had been identified from a registry of patients assessed for treatment with an interdisciplinary pain management program (PMP) at a tertiary National Health Service hospital in northern England (The Walton Centre). All patients had consented for their names to be entered into this registry; the consenting rate for entry is 98%.

Patients were approached for the phenotyping study by letter, and interested patients attended a single study visit. Inclusion criteria were FMS of more than 1 year’s duration, ACR diagnosis 2010^3^ or 1990^26^ (both were assessed on the day, and either qualified), age greater than 18 years, and an average weekly pain intensity of ≥4/10 on a numerical rating scale (NRS) (where 0 = “no pain” and 10 = “pain as bad as you can imagine”). The examination for tender points as per ACR 1990^26^ was conducted after some training had occurred to achieve a pressure of approximately 4 kg/cm^2^. Exclusion criteria included pregnancy or breastfeeding and inadequate understanding of the English language.

With regard to aftersensations, drawing from clinical experience, we expected that patients would find both the clinical examination of tender points^26^ and examination with the pressure algometer (see below) painful for a prolonged period after the assessment, and we wished to study the duration of that response. Patients were given a pain diary to enter the presence or absence of any lingering pain from the examination of ACR 1990 tender points^26^ and algometry test sites on each of days 1 to 5 after their study visit, and these scores were communicated over the telephone after this period.

Upon examination with brushstroke, as part of a protocol to assess pleasant touch, initial patients in this phenotyping study unexpectedly reported post-examination aftersensations, which often seemed unpleasant. These initial research subjects further advised that it was challenging for them to clearly identify aftersensations as “painful” and that the term “uncomfortable” would best encompass the unpleasant sensation that they experienced. We consequently adapted the study protocol to allow a more in-depth assessment of this phenomenon. We obtained ethics approval to inquire about the character of these sensations and to include a pain-free healthy control (HC) comparator group, which was subsequently recruited from university and health care staff.

The phenotyping study received ethics approval from Health and Care Research Board Wales: 18/WA/0234. All participants gave written consent, and they were reimbursed expenses up to £30 for their travel and HCs an additional £30 for their time.

### Study Procedure

After consent and confirmation of eligibility (see above), patients were asked questions pertaining to their general health and fibromyalgia symptomatology; this included the tissue location of their perceived most intense pains in “bones,” “skin,” “muscles,” or “joints,” with multiple answers permitted. Participants also self-completed a set of standardized questionnaires, which were then checked for completeness by a member of the team. These included the EQ-5D^27^, the McGill Short Questionnaire^28^, a Brief Pain Inventory (BPI)^29^, the Hospital Anxiety and Depression Scale (HADS)^30^, the Pain Catastrophizing Score^31^, the Experiences in Close Relationship Questionnaire (Revised) (ECR-R)^32^, the Pain Self-Efficacy Questionnaire (PSEQ)^30^, the Revised Fibromyalgia Impact Questionnaire (FIQR)^33^, and PainDETECT.^34^ All patients were then examined for their skin sensitivity (see next section).

### QST Procedure

A brief mechanical QST protocol was designed to test patients’ mechanical pain threshold and skin sensitivity and was based on the protocol previously published by Boehme et al.^35^ The procedure was performed by RB and AG after training by AM. The tests took place in a quiet, temperature-controlled (21°C) test room.

#### Pressure Pain Threshold

Subjects were asked to sit comfortably, and a standardized script was read to them (see Supplementary Data Figure S2). The script read:

*“I will press this pressure measuring device against one of your muscles. Please immediately say NOW as soon as the usual sensation of pressure changes to an additional sensation which is painful such as ‘burning*,*’ ‘stinging*,*’ ‘drilling’ or ‘aching’. This is not an endurance test, tell us as soon as this becomes painful. This will be carried out a total of 3 times.”*

Static blunt pressure pain threshold was measured with a pressure algometer (FDN200; Wagner Instruments, Greenwich, CT, USA) with a 1-cm^2^ rubber tip, which was placed on the skin. A continuous ramp of increasing intensity (approximately 0.5 kg/s, corresponding to 50 kPa/s with a metronome) was applied until the patient confirmed that the sensation of pressure had changed to an additional one of pain. The patient was not able to see the dial. The pressure pain threshold was determined as the arithmetic mean of three consecutive readings. The test sites were the lateral right arm over brachioradialis and the left leg over vastus lateralis.

#### Brushstroke

Keen to explore gentle touch in greater depth than can be achieved by formal QST, which measures only dynamic mechanical allodynia^11^, we chose to focus on slow and fast brushstroke QST, coupling this with the qualitative perception of skin sensation. This was interrogated through a mixed-methods approach.^36^ Subjects were asked to sit comfortably with their left arm supinated on a pillow. Participants were shown the numerical scales before brushstroke examination and told that they would be stroked with a soft brush. A 30-cm ruler was placed securely alongside their arm. The ruler placement marked the test site and was consistent for all tests. Participants received gentle stroking touch applied manually to the skin of the lateral left forearm in the supinated position at slow (3 cm/s) and fast (30 cm/s) speeds from proximal to distal (with the hair growth) over a 10-cm length. A QST brush (SENSELab Brush-05, Somedic SenseLab AB, Norra Mellby, Sweden) was used to deliver the stimulus and a metronome used to ensure correct speeds. The brushstroke was not obscured from the participants. Immediately after each brushstroke, subjects were asked to rate their perceptions on a grounded 5-cm NRS for pleasantness, intensity, ticklishness, and pain. The anchoring statements are shown in parentheses and were as follows: Pleasantness was rated from –5 (“very unpleasant”) to +5 (“very pleasant”). Participants were then asked to rate the intensity on a grounded NRS of 0 (“no sensation”) to 10 (“very intense”). Ticklishness was rated from 0 (“not ticklish”) to 10 (“very ticklish”), and pain from 0 (“none”) to 10 (“worst possible”).

After the protocol adaptation from participant eight onward (see above), subjects were additionally asked (after completion of these NRS descriptions; ∼30 seconds) about the presence of any brushstroke test sensations that occurred immediately after the cessation of the final stimulus, via a combination of yes/no, multiple-choice, and open-ended questions.^36^ First, the subjects were asked, “After the brushstroke tests, have you felt any lingering sensation?” (“yes” or “no”). If the subjects confirmed the presence of such lingering sensations, they were asked to describe their quality: “Did this lingering sensation feel like” … “pins and needles,” “burning,” or “other”? Subjects were encouraged to report in free text all they felt and were permitted to select or record multiple types of sensations, as relevant. Subjects were then asked whether their sensations after brushstroke were uncomfortable (“yes” or “no”). We have termed sensations extending beyond the brushstroke tests *aftersensations*. For testing protocols, please see Supplementary Data Figure S2.

### Statistics

Data were collated with Microsoft Excel version 16 (Microsoft Corporation, Redmond, WA, USA) and analyzed with GraphPad Prism version 9 (GraphPad Software Inc., San Diego, CA, USA). Normality of data were tested with the Kolmogorov-Smirnov test. For normally distributed data, a paired or unpaired Student *t* test with Welch’s correction for unequal variance was used. For non-normally distributed data, a Wilcoxon matched-pairs rank-sum test was used for paired data, and a Mann-Whitney test was used for unpaired data. For comparison of multiple non-normally distributed groups, a Kruskal-Wallis test was implemented. For categorical binary data, a Fisher’s exact test was used. For the non-normally distributed brushstroke tests and pressure pain threshold data, a Kruskal-Wallis test was used, with a Dunn’s post hoc test between groups with correction for multiple comparisons. A multiple linear regression model using a least-squares approach was used to analyze variability in brushstroke pleasantness, modeling for intercept and main effects only, and residual plots were assessed for assumption validity. Correlation was tested with a Spearman’s correlation coefficient, and again, residual plots were assessed. Odds ratios were calculated for the probability of experiencing aftersensations with FMS. Statistical significance was set at *P* < 0.05, and a correction for multiple comparisons was made for each hypothesis (e.g., Bonferroni). All *P* values under 0.05 were displayed for completeness. We speculated that some aspects of the FIQR were particularly relevant in testing the hypothesis that aftersensations had clinical relevance, and therefore we analyzed several FIQR sub-items individually, which ranged from 0 (least impact) to 10 (maximal impact) (Supplementary Data Table S2).

## Results

### Demographics

Demographic data are displayed in Table 1 for all 51 patients and 16 HCs. In the FMS group, there were 46 females with a mean age of 49.4 years (26 to 66 years) and five males with a mean age of 41.6 years (24 to 58 years). Before recruitment, 32 of the 51 patients had completed a comprehensive, 16-day (>100 hours) interdisciplinary PMP, whereas 19 patients had been assessed for the program but were not treated. Patients with FMS were noted to have a higher weight and body mass index than those of HCs. Patients had a mean symptom duration of 10.6 years, and both their resting pain rating and 7-day average pain rating were 7/10. The average HADS scores for anxiety and depression were both 11.7, which is at the clinical threshold for anxiety and depression. The mean Pain Catastrophizing Scale, EuroQual Visual Analog Scale, and FIQR scores were 24.3, 46.6, and 69.9, respectively. Chronic pain preceding widespread pain was common at 80% (41/51).

**Table 1.** Demographics of HCs and patients with FMS and FMS disease characteristics.

	Healthy Pain-Free Subjects		Patients with Fibromyalgia		
	(n = 16)		(n = 51)		
	Mean	95% CI	Mean	95% CI	
Females, %	100%	–	90%	–	n.s.
Age, years	41.2	35.6 to 46.8	48.7	45.7 to 51.6	<0.05
Body mass index, kg/m^2^	25.9 [15]	22.9 to 28.9	33.5 [43]	31.1 to 35.9	<0.01
Height, m	1.64 [15]	1.61 to 1.67	1.65 [47]	1.62 to 1.67	n.s.
Weight, kg	69.3 [15]	62.3 to 76.3	90.0 [47]	83.5 to 96.5	<0.01
Symptom duration, years	–	–	10.6	8.9 to 12.3	–
Current resting pain	–	–	7.0	6.5 to 7.6	–
Pain in prior 7 days	–	–	7.3	6.8 to 7.8	–
HADS-Anxiety rating	–	–	11.7	10.4 to 12.9	–
HADS-Depression rating	–	–	11.7	10.6 to 12.9	–
Pain Catastrophizing Scale rating	–	–	24.3	20.5 to 28.1	–
EuroQual Visual Analog Scale rating	–	–	46.9	41.7 to 52.0	–
FIQR rating	–	–	69.9	65.6 to 74.2	–

Values are displayed as means with 95% confidence intervals (CIs). Where group size varied, this is displayed in square brackets.

Statistical significance was tested with a Mann-Whitney *U* test. Uncorrected *P* values set at 0.05, after Bonferroni correction *P* < 0.01; n.s.= not significant.

### Patients with FMS Are More Sensitive to Mechanical Stimuli than Are HCs and Perceive Less Pleasure from Gentle Touch (Tactile Anhedonia)

**Figure 1.**
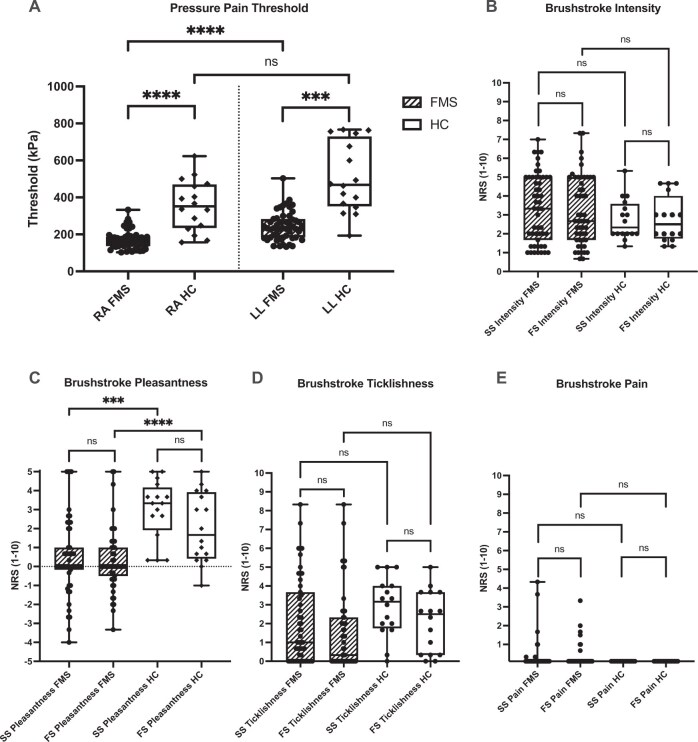
QST results. Box plots display medians, quartiles, and ranges for **(A)** pressure pain threshold and **(B through E)** tactile quantitative sensory testing. SS = slow brushstroke; FS = fast brushstroke; RA = right arm; LL = left leg. For brushstroke tests, statistical significance tested with Kruskal-Wallis with a Dunn’s post hoc test corrected for multiple analyses. ****P < 0.0001; ***P < 0.001; **P < 0.01; *P < 0.05; n.s.= not significant.

Figure 1 displays data from the pressure pain thresholds and the brushstroke tests. As expected, the median pressure pain threshold values were significantly reduced in the patients with FMS at both the right arm and left leg (Figure 1A). Brushstroke intensity (NRS) was not significantly different between the FMS and HC cohorts for either slow or fast brushstrokes (Figure 1B). Patients with FMS reported both slow and fast brushstrokes as significantly less pleasant (Figure 1C). Within the HC cohort, slow brushstrokes were not more pleasant than fast brushstrokes with our multiple-variable analysis (Kruskal-Wallis with Dunn’s post hoc test), which is contrary to reported findings.^35^ We chose, also, to analyze our data as has been done in previous studies that have assessed slow and fast brushstroke QST, in which data were dichotomized by slow or fast brushstroke and tested with a Mann-Whitney *U* test. In this analysis, our data recapitulate the observation previously found (*P* < 0.01).^35^ In our multiple-variable analysis, we found a trend toward reduced ticklishness of brushstrokes in the FMS group, which did not reach significance (Figure 1D). Brushstrokes were rarely painful (i.e., there was little dynamic mechanical allodynia); the median pain intensity during these strokes was zero in both study groups for both slow and fast (Figure 1E). In total, 14% (7/51) of subjects with FMS reported pain scoring between 0.0 and 4.3 out of 10 for slow brushstrokes and between 0.0 and 3.3 out of 10 for fast brushstrokes. No HCs reported pain. Given the small number of patients experiencing dynamic mechanical allodynia (n = 7), there was no trend with regard to this parameter and the perceived anatomic location of the worst pain.

It is conceivable that PMP treatment might alter patients’ affective experiences as a result of the psychological interventions provided as part of the program. Subgroup analysis showed that the slow stroke intensity, slow stroke pain, fast stroke intensity, and fast stroke pain were significantly less in patients who had previously been treated with this intervention; however, after *P* value correction for multiple tests, only slow stroke intensity remained significantly reduced. Interestingly, the brushstroke pleasantness ratings were not affected (Supplementary Data Figure S3).

### Brushstroke Pleasantness Is Not Accounted For By WPI, SSS, PCS, HADS or PainDETECT

**Figure 2.**
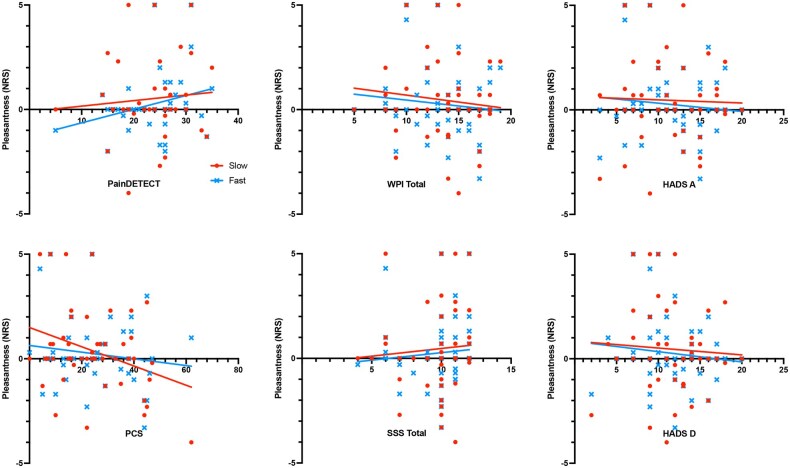
Scatter plots of brushstroke pleasantness against PainDETECT scores. Linear regression lines for average slow brushstroke in red for slow brushstroke and in blue for fast. *Abbreviations:* WPI = Widespread Pain Index; PCS = Pain Catastrophizing Scale; HADS A/B = Hospital Anxiety and Depression Scale Anxiety/Depression. Correlation was tested with Spearman’s correlation coefficient Scale. Significance was set at *P* < 0.05.

We identified a fair degree of variability in the pleasantness ratings in the FMS group. To test for associations that might explain the loss of pleasant touch, we performed a multiple regression analysis with a model considering the degrees of pain-widespreadedness and somatic symptoms (Widespread Pain Index, Symptom Severity Score), psychological factors (Pain Catastrophizing Scale, HADS-Anxiety and HADS-Depression), and neuropathic symptoms as measured by PainDETECT, assuming a least-squares model. No significant association (*P* < 0.05) was found with slow stroke pleasantness and these variables. A slight positive association was identified between symptom severity and slow and fast stroke pleasantness (*P* < 0.05) in the regression model. However, univariate analyses in (Figure 2), show no significant trend in any of the variables tested against brushstroke pleasantness.

### Aftersensations

**Figure 3.**
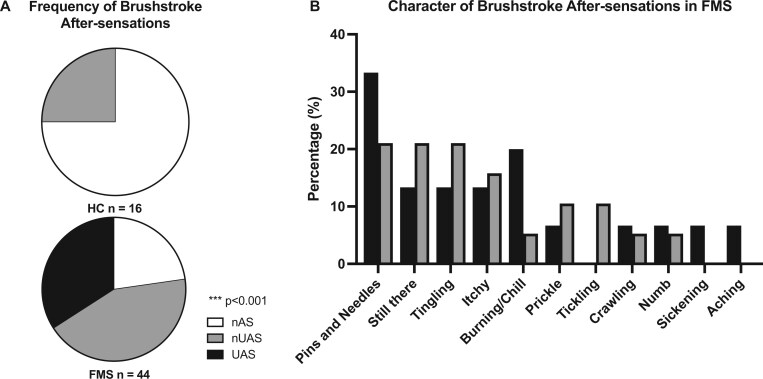
Aftersensations after brushstroke. **(A)** Pie chart showing percentages of subjects reporting sensations at the site of brushstrokes after application of a series of six brushstrokes (three slow and three fast) delivered in an alternating order to the left arm. **(B)** The character of the aftersensations. Patients were able to report any number of sensations. The *P* value pertains to a Fisher’s exact test for the presence of aftersensations between HCs and patients with FMS. Significance was set at *P* < 0.05.

Patients reported lingering pain at the examination sites (tender-point or pressure algometry) with a frequency of 78.1% (25/31) at day 1 and 46.7% (14/30) at day 5. Brushstroke aftersensations were experienced by 77.3% (34/44) of subjects with FMS (Figure 3A). In almost half (15/34) of these patients (15/44 of all tested patients with FMS), the reported sensation was classed as “uncomfortable.” At a 25% (4/10) incidence, HCs experienced aftersensations with a significantly diminished frequency (*P* < 0.0005), and these aftersensations were never uncomfortable. The odds ratio of experiencing aftersensations with FMS was 10.2 (95% confidence interval: 2.5 to 32.4). When only patients who experienced dynamic mechanical allodynia were considered, 83.3% (5/6) had aftersensations, and in 66.8% (4/6), these were uncomfortable.

The qualities of these aftersensations were similar in patients with FMS for both uncomfortable aftersensations (UAS) and non-uncomfortable aftersensations (nUAS), with “pins and needles” being the modal response, followed by a lingering sensation that the brushstroke was still taking place (Figure 3B). The median number of reported sensations was one, ranging from one to three. For HCs, the aftersensations were “cool” (1/4), “brush still there” (1/4), or “tingling” (2/4).

When patients were stratified by whether they had undergone the PMP treatment, no trend was seen in the presence of brushstroke aftersensations. Of the 25 patients who attended the PMP, 76% (19/25) had aftersensations, and of the 19 who did not attend the PMP, 79% (15/19) had aftersensations.

### Patient Phenotypes by Brushstroke Aftersensations

We compared demographics and FMS characteristics between patients with and without brushstroke aftersensations to examine whether this characteristic was associated with a particular clinical phenotype. We found that these groups did not differ in gender, age, FIQR, level of pain, Widespread Pain Index, Symptom Severity Score, pain catastrophizing, or EuroQual Visual Analog Scale (Table 2).

**Table 2.** Descriptive QST (brushstroke) data.

	Brushstroke Aftersensations	No Brushstroke Aftersensations	*P*
Group size	n = 34	n = 10	
Females, %	91.2	100.0	n.s.
Age, years	46.7 (42.3 to 50.6)	49.1 (44.5 to 53.7)	n.s.
Body mass index, kg/m^2^	33.3 (30.1 to 36.4) [31]	38.2 (35.1 to 41.3)	*P* < 0.05
Duration, years	10.3 (8.5 to 12.1)	12.5 (6.5 to 18.5)	n.s.
FIQR Total	71.3 (66.0 to 76.6)	66.6 (54.4 to 78.9)	n.s.
FIQR Level of Pain	7.8 (7.2 to 8.4)	7.9 (7.1 to 8.7)	n.s.
Widespread Pain Index	13.1 (12.1 to 14.1)	15.0 (12.5 to 17.5)	n.s.
Symptom Severity Score	10.1 (9.5 to 10.7)	9.5 (8.1 to 10.8)	n.s.
Slow stroke pleasantness (NRS)	0.2 (–0.5 to 0.9)	1.3 (0.1 to 2.6)	n.s.
Fast stroke pleasantness (NRS)	0.0 (–0.5 to 0.5)	1.1 (–0.1 to 2.2)	*P* < 0.05
Slow stroke pain (NRS)	0.3 (–0.1 to 0.6)	0.2 (–0.2 to 0.5)	n.s.
Fast stoke pain (NRS)	0.3 (0.0 to 0.5)	0.2 (–0.2 to 0.5)	n.s.
Arm pain threshold, kPa	176.1 (158.3 to 194.0)	162.2 (126.5 to 197.9)	n.s.
Leg pain threshold, kPa	245.2 (217.2 to 273.2)	243.7 (190.0 to 297.4)	n.s.
Pain Catastrophizing Scale rating	25.1 (20.3 to 29.9)	20.7 (11.3 to 30.1) [9]	n.s.
EuroQual Visual Analog Scale rating	47.3 (40.9 to 53.7) [33]	52.4 (37.5 to 67.4) [9]	n.s.
PainDETECT	24.3 (21.8 to 26.8) [27]	21.3 (16.8 to 25.9) [6]	n.s.
Lingering pain at 1 day, %	82.3 [19/23]	75.0% [6/8]	n.s.
Lingering pain at 5 days, %	47.8 [11/23]	42.9% [3/7]	n.s.

Pleasantness is displayed on an NRS of –5 [very unpleasant] to 5 [very pleasant]. Pain is displayed on an NRS of 0 to 10. Lingering pain is the pain felt at the examination site after examination. Mean values are represented with 95% confidence intervals in parentheses. Where group size varied, this is displayed in square brackets.

Statistical significance was tested with a Mann-Whitney *U* test. Uncorrected *P* values were set at 0.05; n.s.= not significant. A Bonferroni correction was made for multiple tests in the hypothesis; after 15 tests *P* < 0.003.

Patients were asked about the perceived tissue location of their most intense pains (“bones,” “skin,” “muscles,” or “joints”), with multiple responses permitted. Eleven patients (of n = 44 who completed this question) recorded “skin” as the site of their most intense pain, and those with UAS reported “skin” more often (47%, 7/15) than did the nUAS (20%, 2/10) or nAS groups (2/19, 11%; *P* < 0.05; Supplementary Data Figure S1).

Those patients who experienced aftersensations rated fast brushstrokes significantly more unpleasant than did the other groups (Table 2). Subgroup analyses across the three groups (UAS, nUAS, and nAS) (Supplementary Data Table S1) indicated slow and fast brushstroke pleasantness measures to be significantly different, with the UAS group rating brushstrokes least pleasant compared with the other groups.

Should patients indeed perceive uncomfortable sensations after gentle stroking of their skin, this could well have deleterious manifestations relevant to their everyday lives, such as social interactions within the family. We compared, therefore, the UAS, nUAS, and nAS groups with regard to the relevant FIQR sub-items (Supplementary Data Table S2). No association was found with pertinent individual FIQR measures, e.g., on combing hair, sleep quality, or overall pain, although there appeared to be a trend for the parameter “sensitivity to touch.”

## Discussion

In this study, we have investigated aftersensations persisting beyond the termination of mechanical stimuli applied to the skin of patients with FMS. We found that painful aftersensations (lingering pains) are frequently reported several days after study examination, which included application of blunt pressure during the ACR tender point examination (approximately 4 kg/cm^2^), pressure pain threshold testing, and brushstroke QST. Eighty percent (25/31) of patients had lingering pain on day 1 and 47% (14/30) on day 5 after examination. The existence of this phenomenon after blunt mechanical stimuli has previously been highlighted by others^20^ and has often been communicated by patients in our own clinical practice; however, to our knowledge, our data provide a first account of its prevalence and duration. The pressure required to generate a noxious stimulus in HCs is typically 200–300 kPa for the arm and 250–450 kPa for the leg.^37^ FMS pressure measurements observed in the present study are lower than these, representing mechanical hyperalgesia.

This finding also confirms that tender point examination in FMS can regularly cause long-lasting discomfort. A count of painful tender points was part of the now superseded ACR 1990 diagnostic criteria.^26^ Although the current diagnostic criteria (ACR 2016)^38^ do not include a count of painful tender points, the 1990 criteria are still in wide use, and our results could support diagnosticians in choosing a different appropriate set of diagnostic criteria. These data also support clinical advice to minimize repeat examination in patients with FMS in all settings^39^, as well as our recommendation that patients should be given the opportunity to consent to examination after having been informed that they might expect increased pain from the examination.

To our knowledge, this is the first report of evoked paresthesia or aftersensations after skin-brushstroke testing in FMS. We found that such sensations are common compared with HCs and that patients, but not HCs, often perceive them as uncomfortable. Consistent with the literature, allodynia, defined as pain *during* brushstroke application, was reported by some of our patients (7/51).^37^ This phenomenon in health, however, is not expected to continue after stimulus withdrawal.

Within the FMS group*,* reports of uncomfortable aftersensations, as opposed to non-uncomfortable or no aftersensations, after brushstroke were associated with reports of reduced pleasantness during brushstroke; on average, slow and fast brushstrokes were, in fact, perceived as unpleasant by this group. Experience of uncomfortable brushstroke aftersensations was not associated with blunt pressure sensitivity, however (Supplementary Data Table S1), and it was particularly frequent in those patients who noted that their skin was the location of their worst pain (Supplementary Data Figure S1). These features could, therefore, characterize a distinct FMS clinical subgroup in whom touch processing is differentially affected.

Reduced brushstroke pleasantness (i.e.*,* during the brushstroke; termed *anhedonia*), as detected in our study, is consistent with recent reports by Boehme et al.^35^ and others, although its cause is still unclear.^40^^,^^41^ By mapping cortical activity during brushstroking with functional magnetic resonance imaging, Boehme et al. found an inverted pattern of insula activity compared with HCs and inferred, therefore, that FMS anhedonia might be related to aberrant central nervous system evaluative processing.^35^ Nonetheless, the finding of anhedonia in FMS does not exclude the possibility of abnormal signal processing of input from sensory afferents. Certainly, evidence of small-fiber pathology in FMS is mounting^42^^,^^43^, with reports of reduced epidermal nerve fiber density^41^ and reduced vascular innervation.^8^ Bosma et al. showed that painful aftersensations after thermal stimuli are associated with increased activation in the cervical cord dorsal horn in patients with FMS^23^, pointing perhaps to continued afferent activity.

We expected to see an inverse correlation of brushstroke pleasantness in our FMS cohort with the PainDETECT score, the lack of this, therefore, may argue against small-fiber pathology as one mechanism of anhedonia in FMS. However, should this be true, and brushstroke testing does predict small-fiber pathology, then using brushstroke testing to diagnose small-fiber pathology would be much preferable to a painful skin biopsy.^42^ In summary, anhedonia could be a consequence of abnormal afferent input, altered spinal processing, or altered brain processing.

It is tempting to speculate that at least the “early” (i.e., ∼15 seconds^20^) brushstroke aftersensations might be mediated by slowly conducting C fibers. One such candidate is the CT fiber, a low-threshold afferent that discharges maximally with a slow (1–10 cm/s) brushstroke and elicits a pleasant sensation in subjects.^44^ These CT fibers, which are suited to respond to a “gentle caress,” are thought to provide the “neurobiological substrate for the affective and rewarding” aspects of touch.^45^ This “social” touch is clearly important for physical and social well-being^45^, and its loss, tactile anhedonia, is a clear feature of FMS.^35^ Because CT fibers fire preferentially after stimuli with brushstroke speeds of 1–10 cm/s, one might expect aftersensations to be more strongly associated with slow rather than fast brushstroke pleasantness. However, we note that this would suppose that CT firing is normal in FMS—an assumption that has yet to be confirmed. CT afferent firing in health has a propensity to exhibit afterdischarges upon cessation of an innocuous tactile stimulus^9^, which further points to a potential role in the development of aftersensations. A further way to investigate this would be a comparison between brushstrokes and vibro-tactile stimuli, the latter of which strongly activate A-fiber low-threshold mechanoreceptor afferents but only weakly excite CT afferents.^9^^,^^45^ However, it is important to note that brushstroking will activate A-beta and A-delta fibers, as well as the CT afferents, which could all integrate centrally. There are, of course, other explanations, such as reverberating circuits in the central nervous system or the aforementioned afterdischarging afferents.

Intriguingly, patients who had attended our PMP treatment had reduced slow brushstroke intensity ratings. Brushstroke pleasantness ratings were not altered, however. These data might provide the first evidence of PMP treatment potentially affecting selected sensory characteristics in FMS. However, given that all patients in this study had been assessed for the suitability of PMP treatment, it is alternatively possible that the entrance criteria for our PMP also selected for patients with a distinct sensory phenotype. A prospective study in PMP participants would shed more light on these issues.

Dynamic mechanical allodynia and dysesthesias are noted in several neuropathic pain conditions (e.g., post-herpetic neuralgia pain)^46^, as well as in fibromyalgia.^16^ We note that dynamic mechanical allodynia was also present in our study at a 10% incidence. The cohort of patients with aftersensations also had a higher incidence of dynamic mechanical allodynia.

The PainDETECT questionnaire was validated as a tool to predict the relative contribution of neuropathic pain in neuropathic and nociceptive (e.g., osteoarthritis) conditions. Patients with fibromyalgia were excluded from the validation process, although PainDETECT has been increasingly used in this population. It has been proposed that fibromyalgia should not be considered a neuropathic pain state^47^; however, with mounting evidence that FMS has a peripheral component^5–9^, it is perhaps unsurprising that patients with fibromyalgia experience similar sensory phenomena as patients suffering from neuropathic conditions.^47^ In this study, it is curious that tactile anhedonia was not associated with the PainDETECT score, fibromyalgia severity (FIQR), measures of “widespreadedness” (Widespread Pain Index, Symptom Severity Score), or cognitive qualities, such as pain catastrophizing. Regardless of the underlying mechanism, be this neuropathy or something else, it seems plausible that tactile anhedonia points to a distinct phenotype.

The German Research Network on Neuropathic Pain (DFNS)^11^ has generated a robust and reproducible QST protocol for assessing patients with neuropathic pain. Though a thorough, useful, and widely accepted approach, the experimental protocol does not account for the assessment of sensations that linger after stimulus withdrawal. We suggest, therefore, that the DFNS protocol might have been failing to capture a common and germane clinical sequela of chronic pain.

Our present study is limited, as it was not designed to interrogate aftersensations in detail, which were a surprise finding. We did not record their duration or examine whether their qualities were the same for both slow and fast brushstrokes. It should be noted that we did record a significant reduction in pleasantness between slow and fast brushstrokes in HCs and not in patients with FMS, which has been previously noted and underpins the C-fiber tactile theory, although it failed to meet significance in our study in all analyses. The control group was small and was restricted to females, and it did not, therefore, match the FMS group in this regard. Although the proportion of male patients with FMS included was small (n = 3), it is conceivable that aftersensations had some gender bias: In our FMS cohort, all males for whom we have data had aftersensations. The HC group also differed by age, body mass index, and weight, which were potential confounders.

It is important to note that in the present study we measured both brushstroke QST and pressure algometry, and we performed tender point examination. It is not possible, therefore, to extract the relevant contributions of each of these in relation to persisting aftersensations (lingering pain). We further note that the pressure algometry, unlike the tender point examination, stopped exactly at the point of painfulness and involved two points on the body (the tender point examination involved 18 points). Therefore, we assume that the tender point examination was responsible for most of the lingering pain; however, this study was not designed to confirm this.

We did not record data on the presence or absence of aftersensations in the HC group after pressure pain threshold detection, and this is a clear limitation. HCs have been reported to experience painful aftersensations after blunt pressure mechanical stimulus, though the frequency was low at 12% and of an unknown duration.^25^ Patients with FMS are probably heterogenous in sensory phenotypes^48^, and it is true that such variability would hamper our ability to distinguish between groups. As this was an exploratory study, we were not powered to tease out subtle relationships between aftersensations and psychometric data. A larger study is warranted for this. Our cohort was obtained from a tertiary referral center and might not, therefore, have been fully representative of all patients in the community. With these pilot data, it would now be possible to adequately power a study to test the hypothesis that the uncomfortable aftersensation group is more sensitive (as suggested by the association with reduced brushstroke pleasantness), possibly with respect to social measures, such as assessed by the Touch Experiences and Attitudes Questionnaire.^49^ In addition to these measures, biochemical changes might also be investigated—for example, cortisone or endorphin levels locally or systemically. We placed emphasis on the bothersomeness of patient symptomatology, and, in this regard, further work might look to investigate the presence and character of itch and how this is related to ticklishness. Examinations for atypical allodynia and paresthesia can easily be done in the clinical setting, and understanding patient phenotypes in terms of these could be clinically useful.

## Conclusion

Here we report, for the first time, the presence of aftersensations after the cessation of an innocuous light touch stimulus in a cohort of patients with fibromyalgia. When present, these aftersensations are often uncomfortable. We also report on the prevalence of lingering pain after pressure examination. We suggest that these findings have key implications for clinicians examining patients and ramifications for experimental trajectories. The recognition of these aftersensations could reassure patients and inform the advice and education given to them. Further research is, of course, needed to investigate these phenomena in FMS and other chronic pains. In this regard, we note that medical efficacy is contingent on placing patients at the center of diagnostic formulations and the interrogation of etiologies.

## Supplementary Data

Supplementary Data may be found online at http://painmedicine.oxfordjournals.org.

## Supplementary Material

pnaf080_Supplementary_Data
