# Supplementary material for: Aftersensations and Lingering Pain After Examination in Patients with Fibromyalgia Syndrome
Source: Pain Med. 2025 Aug 6;26(9):618–27. doi: 10.1093/pm/pnaf080 (PMC12405746; doi:10.1093/pm/pnaf080)
Supplement: pnaf080_Supplementary_Data [file pnaf080_supplementary_data.docx]

**Supplementary Material**

***Table S1***

|  | **Uncomfortable Brushstroke After-sensations (UAS)** | ***Non-uncomfortable* Brushstroke After-sensations**  **(nUAS)** | **No Brushstroke After-sensations (nAS)** |  |
| --- | --- | --- | --- | --- |
| **Group size** | n = 15 | n = 19 | n = 10 |  |
| **Females (%)** | 93.3 | 89.5 | 100.0 |  |
| **Age (years)** | 45.4 (39.4– 51.5) | 47.6 (41.9 – 53.4) | 49.1 (44.5 – 53.7) |  |
| **BMI (kg/m2)** | 31.9 (25.9 – 37.8) [12] | 34.3 (30.4 – 38.1) [16] | 38.2 (35.1 – 41.3) [8] |  |
| **Duration (years)** | 10.5 (7.5 – 13.5) | 10.1 (7.7 – 12.5) | 12.5 (6.5 - 18.5) |  |
| **FIQR Total** | 68.7 (59.8 – 77.6) | 73.4 (66.4 – 80.4) | 66.6 (54.4 – 78.9) | n.s. |
| **FIQR Level of Pain** | 7.6 (6.8 – 8.5) | 7.9 (7.0 – 8.8) | 7.9 (7.1 – 8.7) | n.s. |
| **FIQR Sleep quality** | 8.3 (6.8 – 9.9) | 8.6 (7.7– 9.6) | 8.3 (6.6 – 9.9) | n.s. |
| **FIQR Touch** | 6.7 (5.3 – 8.1) | 7.3 (6.2 – 8.4) | 5.6 (3.8 – 7.4) | n.s. |
| **FIQR Brush** | 5.3 (3.6 – 7.0) | 4.9 (3.3 – 6.6) | 4.2 (2.3 – 6.1) | n.s. |
| **WPI** | 13.7 (12.3 – 15.0) | 12.6 (11.1 – 14.2)) | 15.0 (12.5 – 17.5) | n.s. |
| **SSS** | 9.9 (9.2 – 10.7) | 10.2 (9.2 – 11.1) | 9.5 (8.1 – 10.8) | n.s. |
| **Slow stroke pleasantness (NRS)** | -0.8 (-1.8 – 0.1) | 1.0 (0.9 – 1.9) | 1.3 (0.1 – 2.6) | p<0.015 |
| **Fast stroke pleasantness (NRS)** | -0.8 (-1.3 - -0.2)) | 0.6 (-0.1 – 1.2) | 1.1 (-0.1 – 2.2) | p<0.0022 |
| **Slow stroke pain (NRS)** | 0.6 (-0.2 -1.3) | 0.0 (0.0 – 0.1) | 0.2 (-0.2 – 0.5) | n.s. |
| **Fast stoke pain (NRS)** | 0.6 (0.0 - 1.2) | 0.0 (0.0 – 0.0) | 0.2 (-0.2 – 0.5) | n.s. |
| **Arm pain threshold (kPa)** | 168.1 (148.6 – 187.6) | 182.5 (153.0 – 211.9) | 162.2 (126.5 – 197.9) | n.s. |
| **Leg pain threshold (kPa)** | 251.8 (199.9 – 303.7) | 240.0 (206.2 – 273.8) | 243.7 (190.0 – 297.4) | n.s. |
| **PCS** | 26.3 (18.2 – 34.5) | 24.0 (17.6 – 30.4) [18] | 20.7 (11.3 – 30.1) [9] | n.s. |
| **EQ-VAS** | 44.0 (36.0 – 52.1) | 49.9 (39.9 – 60.0) | 52.4 (37.5 – 67.4) [9] | n.s. |
| **Pain DETECT** | 25.0 (21.5 – 28.5) [12] | 23.7 (19.8 – 27.5) [15] | 21.3 (16.8 – 25.9) [6] | n.s. |
| **Lingering Pain at 1 Day (%)** | 81.8 [9/11] | 83.3 [10/12] | 75.0% [6/8] | n.s. |
| **Lingering Pain at 5 Days (%)** | 36.4 [4/11] | 58.3 [7/12] | 42.9% [3/7] | n.s. |
| ***Table S1 - Descriptive Quantitative Sensory Testing (Brushstroke) Data***  *Mean values are represented with 95% confidence intervals in parenthesis. If group size varied it is displayed in square brackets. Lingering pain is the pain felt after the examination on top of usual pain. Pleasantness is displayed on a numerical rating scale (NRS) -5 [very unpleasant] to 5 [very pleasant], pain on an NRS of 0 -10 Abbreviations: FIQR = Fibromyalgia impact questionnaire revised; WPI = Widespread pain index; SSS = Symptom severity score; PCS = Pain catastrophizing scale; EQ-VAS = EuroQual visual analogue scale. We reported on FIQR total and relevant sub scores (0-10) where 10 is maximal disability or impact). Statistical significance tested with a Kruskal-Wallis test. Significance set at p<0.05; n.s., not significant.. A Bonferroni correction was made for multiple tests in the hypothesis; after 18 tests p<0.0028).* | | | | |

***Table S2***

|  | **Brishstroke After-sensations (AS)** | **No Brishstoke After-sensations (nAS)** |  |
| --- | --- | --- | --- |
| **Group size** | n = 34 | n = 10 |  |
| **Females (%)** | 91.2 | 100.0 | n.s. |
| **Age (years)** | 46.7 (42.3– 50.6) | 49.1 (44.5 – 53.7) | n.s. |
| **BMI (kg/m2)** | 33.3 (30.1 – 36.4) [31] | 38.2 (35.1 – 41.3) | p<0.05 |
| **Duration (years)** | 10.3 (8.5 – 12.1) | 12.5 (6.5 - 18.5) | n.s. |
| **WPI** | 13.1 (12.1 – 14.1) | 15.0 (12.5 – 17.5) | n.s. |
| **SSS** | 10.1 (9.5 – 10.7) | 9.5 (8.1 – 10.8) | n.s. |
| **FIQR Total** | 71.3 (66.0 – 76.6) | 66.6 (54.4 – 78.9) | n.s. |
| **FIQR Level of Pain** | 7.8 (7.2 – 8.4) | 7.9 (7.1 – 8.7) | n.s. |
| **FIQR Sleep Quality** | 8.5 (7.7 – 9.3) | 8.3 (6.6 – 9.9) | n.s. |
| **FIQR Energy** | 7.0 (6.0 – 8.0) | 7.8 (6.1 – 9.5) | n.s. |
| **FIQR Touch** | 7.1 (6.2 – 7.9) | 5.6 (3.8 – 7.4) | n.s. |
| **FIQR Brushing** | 5.1 (4.0 – 6.2) | 4.2 (2.3 – 6.1) | n.s. |
| ***Table S2 – Descriptive Quantitative Sensory Testing (Brushstroke) Data (ACR and FIQR Sub-categories)***  *Mean values are represented with 95% confidence intervals in parenthesis. Where group size varied this is displayed in square brackets. Abbreviations: ACR = American College of Rheumatology; FIQR = Fibromyalgia impact questionnaire revised; WPI = Widespread pain index; SSS = Symptom severity score. We reported on FIQR total and relevant sub scores (0-10) where 10 is maximal disability or impact). Statistical significance tested with a Mann Whitney U test. Significance set at p<0.05; n.s., not significant.* | | | |

***Figure S1***

***Figure S1: Where is The Worst Pain?***

*Patients were asked where their pain was the worse. They could choose any number of responses from’ skin’, ‘muscles’, ‘bones’ or ’joints’ with multiple answers permitted.*

***Figure S2***

******

******

***Figure S2: Protocols for Quantitative Sensory Testing***

***Figure S3***

******

***Figure S3: Brushstroke Affective Qualities Stratified by Attendance on the Walton Pain Management Course***

*Boxplots display medians, quartiles, and ranges. Abbreviations: SSS = slow brushstroke pleasantness; SSI = slow stroke intensity; SST = slow stroke ticklishness; SSP = slow stroke pain; FSS = fast brushstroke pleasantness; FSI = fast stroke intensity; FST= fast stroke ticklishness; FSP = fast stroke pain; PMP = pain management program. ‘NO’ or ‘YES’ refer to attendence on the PMP. Statistical significance assessed with Mann Whitney U tests for unpaired data. **** p<0.0001; ** p<0.01; * p<0.05; n.s., not significant; Bonferroni corrections for multiple tests were made for 8 comparisons in total (p<0.00625).*
